# A low technology emanator treated with the volatile pyrethroid transfluthrin confers long term protection against outdoor biting vectors of lymphatic filariasis, arboviruses and malaria

**DOI:** 10.1371/journal.pntd.0005455

**Published:** 2017-04-07

**Authors:** Sheila B. Ogoma, Arnold S. Mmando, Johnson K. Swai, Sebastian Horstmann, David Malone, Gerry F. Killeen

**Affiliations:** 1 Ifakara Health Institute, Environmental Health and Ecological Sciences Thematic Group, Coordination Office, Dar es Salaam, United Republic of Tanzania; 2 US National Research Council, National Academies of Sciences, Engineering and Medicine, Washington, D.C., United States of America; 3 Bayer CropScience Aktiengesellschaft, Monheim, Germany; 4 Innovative Vector Control Consortium, Liverpool School of Tropical Medicine, Liverpool, United Kingdom; 5 Liverpool School of Tropical Medicine, Vector Biology Department, Liverpool, United Kingdom; The Connecticut Agricultural Experiment Station, UNITED STATES

## Abstract

**Background:**

The vapor phase of the volatile pyrethroid transfluthrin incapacitates mosquitoes and prevents them from feeding. Although existing emanator products for delivering volatile pyrethroids protect against outdoor mosquito bites, they are too short-lived to be practical or affordable for routine use in low-income settings. New transfluthrin emanators, comprised simply of treated hessian fabric strips, have recently proven highly protective against outdoor-biting vectors of lymphatic filariasis, arboviruses and malaria, but their full protective lifespan, minimum dose requirements, and range of protection have not previously been assessed.

**Methodology:**

The effects of transfluthrin-treated hessian strips upon mosquito biting exposure of users and nearby non-users, as well as dependence of protection upon treatment dose, were measured outdoors in rural Tanzania using human landing catches (HLC).

**Principal findings:**

Strips treated with 10ml of transfluthrin prevented at least three quarters (p < 0.001) of outdoor bites by *Anopheles arabiensis*, *Culex* spp. and *Mansonia* spp. mosquitoes, and >90% protection against bites on warmer nights with higher evaporation rates, for at least one year. Strips treated with this high dose also reduced biting exposure of non-users at a distance of up to 5m from the strips for *An*. *arabiensis* (p < 0.001) and up to 2m for *Mansonia* spp. (p = 0.008), but provided no protection to non-users against *Culex* spp. No evidence of increased risk for non-users, caused by diversion of mosquitoes to unprotected individuals, was found at any distance within an 80m radius. A dose of only 1ml provided equivalent protection to the 10ml dose against *An*. *arabiensis*, *Culex* spp. and *Mansonia* spp. mosquitoes over 6 months (p < 0.001).

**Conclusions/Significance:**

Transfluthrin-treated hessian emanators provide safe, affordable, long-term protection against several different pathogen-transmitting mosquito taxa that attack humans outdoors, where they are usually active and cannot be protected by bed nets or residual sprays with conventional, solid-phase insecticides.

## Author summary

Many vector-borne parasites and arboviruses are transmitted by outdoor-biting mosquitoes, especially in the evenings and mornings, often despite high coverage of houses with insecticidal nets and residual sprays. The goal of the study was to measure the long-term durability of protection provided by a new, low-technology, passive emanator for the volatile pyrethroid transfluthrin against outdoor-biting mosquitoes. Treated hessian strips reduced exposure of users to three different types of vector mosquitoes by more than three quarters over the first year after treatment, and prevented more than 90% of bites on warmer nights when the active ingredient evaporated faster. Treated strips protected non-users against some vector mosquito species, as far away as 5m from the strip. Reassuringly, mosquitoes were not diverted to nearby unprotected individuals anywhere within an 80m radius. Transfluthrin-treated hessian strips may have considerable potential for integrated control of multiple vectors of filariasis, arboviruses and malaria, which are co-endemic in many tropical areas.

## Introduction

Long lasting insecticidal nets (LLINs) and indoor residual spraying (IRS) are used extensively to control transmission of malaria, lymphatic filariasis, and several arboviruses by indoor-biting mosquitoes. Across much of the tropics, two or more mosquito-borne pathogens are co-endemic and are often transmitted by the same vectors [1]. These tools are effective against anthropophilic (prefer feeding on humans), endophagic (feed indoors) and endophilic (rest indoors) mosquitoes [2,3] but are less effective against exophagic (feed outdoors) and exophilic (rest outdoors) mosquitoes [4]. A wide diversity of outdoor-biting mosquitoes mediate transmission of pathogens that cause filariasis, dengue, yellow fever and malaria, frustrating efforts to eliminate them with LLINs and IRS [5]. Additional vector control tools are therefore clearly required to simultaneously target the many different species of vector mosquitoes that bite humans outdoors and mediate transmission of several neglected tropical diseases.

The most effective pyrethroid insecticides are applied to solid surfaces, such as house walls and ceilings (IRS) or bed nets (LLINs), where mosquitoes may be exposed to them when they make physical contact with those surfaces. However, some less polar insecticides, such as the fluorinated pyrethroids, like metofluthrin and transfluthrin, have lower melting points hence can melt into liquids at ambient temperatures in the tropical areas and are slightly volatile so they release vapor into the air. Low doses of airborne pyrethroids induce sub-lethal responses such as: repellency, deterrence, feeding inhibition and reduce fecundity as opposed to higher doses of contact insecticides that induce knock down and mortality of mosquitoes. Nevertheless, these sub-lethal responses are likely to decrease vectorial capacity of mosquitoes and consequently reduce disease transmission that could be equivalent to toxic contact insecticides [6]. Vapor-phase insecticides are typically formulated as coils, emanators or candles [7]. Transfluthrin [8] and metofluthrin [9] mosquito coils have been shown to prevent malaria infection, notably in combination with LLINs [8]. However, the effectiveness of these products is attenuated by the need for regular compliance by users, reapplication or retreatment of treated clothing or other substrates [10,11], or active electrical energy input, which may be impractical in many rural tropical settings [12]. Passive emanators for releasing such vapor-phase active ingredients have been evaluated against outdoor-biting mosquitoes [13,14,15,16]. These tools do not require heating or electricity and may require minimal compliance by users if the active ingredient is released into the surrounding air, to protect a space around the user. Cost-effectiveness of existing gold standard personal protection measures against mosquitoes is typically limited by product durability. The longest-lasting passive metofluthrin emanator products we are aware of reduced outdoor biting rates of *Culex quinquefasciatus* mosquitoes for up to 8 weeks, after which protection declined rapidly [14]. No evidence has been published that any of these factory-formulated, disposable products can provide high levels of outdoor protection for longer periods, whereas LLINs can protect sleeping spaces for years at a time.

A simple low-technology emanator has been developed, which consists of a strip of hessian sacking hand-treated with transfluthrin possibly allowing repeated retreatment. This emanator is shown to have high efficacy for reducing exposure to mosquito bites for up to six months [17,18]. However, neither of these exploratory studies was conducted long enough to determine the full durability of the prototype, the minimum dose required, or rigorously assessed potential for diversion to nearby non-users. In order to more convincingly assess the full potential of transfluthrin-treated hessian strips as a protection measure against outdoor-biting mosquitoes, this study was undertaken to measure their full long-term protective efficacy over more than two years and assess the protective efficacy of different doses of transfluthrin. The spatial activity of transfluthrin-treated strips was also quantified by measuring the distance over which protection extended and determine whether or not nearby non-users were at a higher risk of being bitten by mosquitoes diverted from users. The effect of changes in temperature on efficacy of strips was also investigated.

## Methods

### Study area

**Fig 1:**
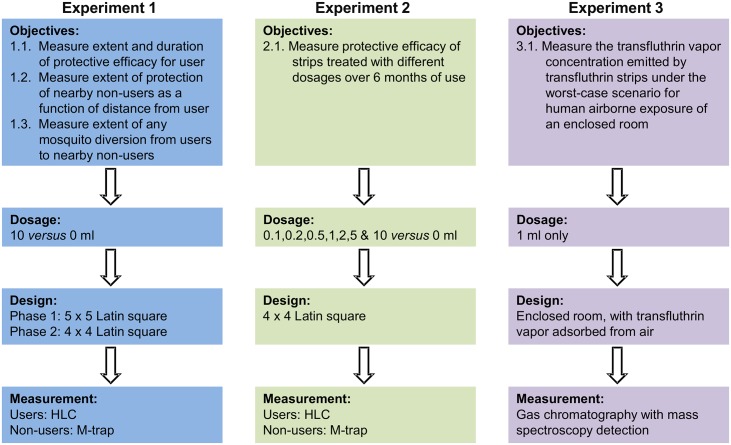
A schematic illustration of key features and work flows for the three experiments comprising this study.

The field study described herein was conducted in the Kilombero Valley of southern Tanzania. Several species of *Culex*, *Anopheles*, and *Mansonia* mosquitoes including several known vectors of not only malaria [6,19,20,21], but also filariasis, and five different arboviruses [22] have been reported in this locality. Since scale up of LLINs in 2008, *Anopheles gambiae* sensu stricto population densities have declined, leaving exophagic *An*. *arabiensis* mosquitoes as the most abundant human-biting *Anopheles* species [23,24], alongside substantial populations of *Ma*. *africana*, *Ma*. *uniformis*, *Cx*. *quinquefasciatus*, *Cx*. *univittatus* and *Cx*. *theileri*. While the physiological resistance status of these various culicines is unknown, local populations of *An*. *arabiensis* have become resistant to pyrethroids (permethrin, lambda-cyhalothrin and deltamethrin) by 2014 [20] when the final year of long-term efficacy testing and the dose-response evaluations described below were conducted (Fig 1, Experiments 1 and 2, respectively). This area typically experiences two rainy seasons every year: intermittent, sporadic rains from November to January and steadier, more consistent rainfall from March to May. Annual rainfall ranges between 1200 and 1800 mm and annual mean temperature ranges between 20°C and 32°C.

### Formulation of transfluthrin—Treated strips

Rectangular strips of hessian fabric, measuring 4 m by 30 cm were cut out of jute (*Corchorus capsularis*) gunny bags that had been imported from India. Strips were treated with different doses of 97% technical grade transfluthrin (Shenzhen Sunrising Industry Company, China) as previously described [17,18]. Each strip was soaked in an emulsion of 0.1, 0.2, 0.5, 1, 2, 5 or 10 ml of transfluthrin, 90 ml of Axion liquid detergent (Orbit Chemical Industries Ltd, Nairobi and Colgate-Palmolive East Africa Ltd) and 400 ml of water and then left to dry indoors overnight at room temperature. Control strips were soaked in an equivalent mixture of water and Axion liquid detergent.

### Study design

The study comprised three distinct experiments to (1) determine the long-term protective efficacy of transfluthrin-treated Hessian strips, (2) determine how their efficacy varies with application dose, and (3) determine the maximum transfluthrin vapor concentration a human user of such treated strips could be exposed to (Fig 1).

### Experiment 1: Long-term protective efficacy of transfluthrin treated strips

This study was conducted in two phases. The first phase lasted almost one full year post-treatment, from 25^th^ of July 2012 until the 6^th^ of June 2013. The second phase spanned the period from the last quarter of the second year post-treatment, to slightly beyond two and a half years post treatment, from the 30^th^ of April 2014 until the 1^st^ of January 2015.

**Fig 2:**
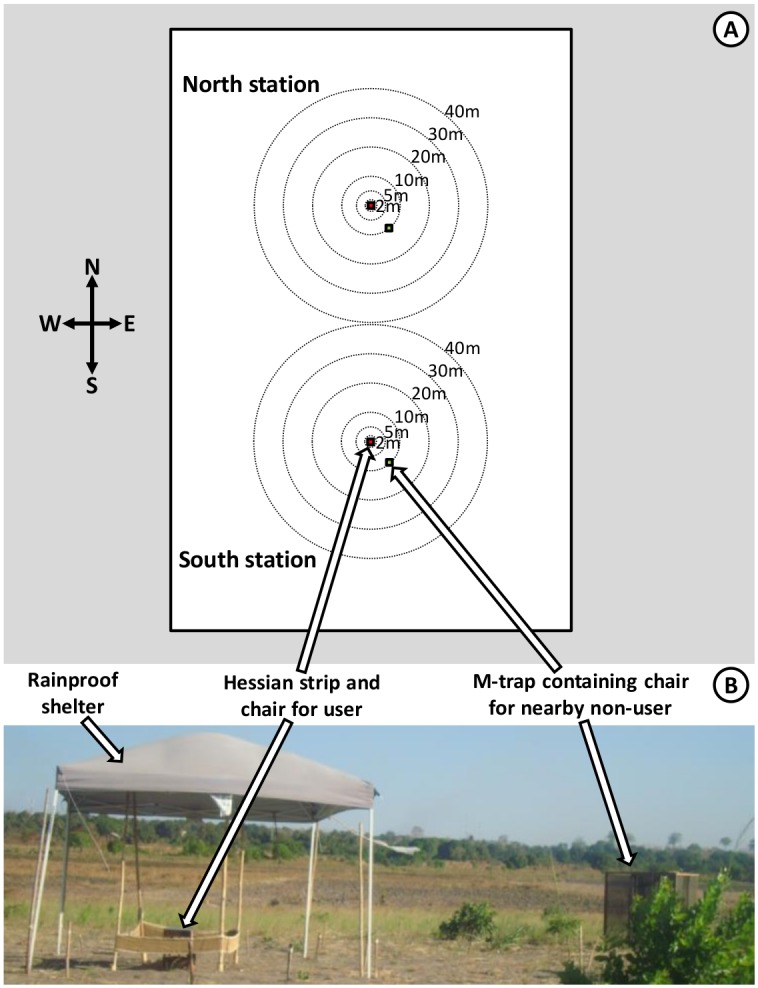
Schematic illustration of the design of experiments 1 and 2. Schematic (**A**) and photographic (**B**) illustration of the arrangement of human landing catches (HLC) by users of treated or untreated sacking strips and non-users sitting in exposure free M traps at specific randomized distances and angles at which a human baited trap was placed. The HLC captures conducted by the users of treated and untreated strips were used to quantify personal protection provided by the treated strips (Fig 1, Objective 1.1). Captures with nearby M traps occupied by participants lacking any treated or untreated strip were used to quantify the degree to which proximity to a protected strip user might decrease (Fig 1, Objective 1.2) or increase (Fig 1, Objective 1.3) biting exposure, over a range of distances varying from 2 to 40 meters. The same phenomenon was assessed at a distance of 80 meters by comparing the number of mosquitoes caught by users of untreated strips on nights when the other user at the other catching station in the same site used a treated strip with the numbers caught when the nearby strip user had an untreated strip.

In the first phase, (Fig 1) five strips treated with 10ml of technical grade transfluthrin (first emulsified by mixing with 90 ml of water and 10ml of liquid detergent as described above) and five untreated control strips were simultaneously assessed by rotation through four separate open-field sites (Fig 2A) within the village of Lupiro (8.385°S and 36.670°E). In this first phase, treated and untreated strips were moved in pairs between field sites and the semi-field tunnel every 3 consecutive experimental nights (See below for details of the three-night randomization cycle for strip treatments) following a 5×5 Latin square design, spanning 15 nights of experimentation for each round of experimental replication. In order to allow volunteers and supervisors four nights per working week to sleep normally, each 3-night cycle of treatment randomizations was allocated to a single working week, so each full round of experimentation was distributed across 5 working weeks. Overall, 5 rounds of experimental replication were completed over the course of phase 1, comprising over 75 nights of experimentation, 600 person-nights of outdoor human landing catches (HLC) measurements, and the same number of person-nights of outdoor mosquito catches measured by human-baited M-trap. The M-trap consisted of netting material wrapped around a wooden frame. The M-trap has two compartments, one occupied by a seated male volunteer protected by a netting panel door and another with an entry slit into which mosquitoes can fly, so that they are captured without being able to make contact with the human occupant.

Also, by the time the second phase (Fig 1) was initiated, one of the strips had degraded due to fungal growth and was disposed of. Therefore, the four remaining strips were rotated through the same four field sites in Lupiro. Treated and untreated strips were moved in pairs between the four field sites every 3 nights (See below for details of the three-night randomization cycle for strip treatments) following a 4×4 Latin square design, spanning 12 nights of experimentation for each round of experimental replication. For same practical logistical reasons described for phase 1, each treatment randomization cycle of 3 nights was completed within a working week, allowing volunteers four free nights off, so each round of experimental replication was completed over the course of a four-week period. The first round of experimental replication was conducted. The first round of experimental replication was conducted between the 30^th^ of April and 21^st^ of May 2014. While a second round was initiated soon afterwards in early June 2014, it was immediately observed that the stock transfluthrin froze into a solid, and that the strips provided little if any protection, following onset of the cool season. Experiments were therefore terminated before this attempt to conduct a second round of replication was complete, and suspended until the exceptionally long cool season that followed finally abated. Complete second and third rounds of experimentation were therefore conducted between the 27^th^ of October 2014 and the 13^th^ of November, and then between the 15^th^ of December and the 1^st^ of January 2015, respectively. Overall, the three rounds of experimental replication that were completed over the course of phase 2 comprised of 36 nights of experimentation, 144 person-nights of outdoor HLC measurements, and the same number of person-nights of outdoor mosquito catches measured by human-baited M-trap.

Each field site consisted of a cleared, open, rectangular field, measured out as 180m by 120m with the long axis pointing north and south (Fig 2A), located ≥100 meters away from the nearest human residence. The two stations at each particular site, where users sat inside the perimeter of a strip (Fig 2B), were positioned 80m apart and 80m away from the perimeters of the site (Fig 2A). These field sites were located ≥300m from each other, so that they could be considered as separate experimental units. In each site, two strips, treated (T) or untreated control (C) were attached to frames of four wooden poles (Fig 2A) forming an area of 4m^2^ [17].

Over every three sequential nights of experimentation, each of the following three arrangements of strips were randomly allocated to each night, where the treatment status of the strip of the user at a given station is denoted as either untreated controls (C) or transfluthrin-treated (T), with the status of the nearby strip at the other station denoted with a subscript, and the status of the catcher north denoted to the left of the hyphen while that of the south station is to the right: (1) An untreated control strip at the northern station and a treated strip in the southern station (C_T_-T_C_), (2) Untreated control strips at both the northern and southern station (C_C_-C_C_), and (3) A treated control strip at the northern station and an untreated control strip at the southern station (T_C_-C_T_). The treatment arrangement selected was the same across all 4 sites on each night of experimentation. The effects of strip treatment status upon human user exposure to mosquito bites was determined by outdoor HLC, conducted by a single adult male volunteer sitting in a chair within the perimeter of the strip. HLC for this set of experiments were conducted from 18.30 hours each evening until 06:30 hours each morning, broken into 6 sequential periods of 2 hours with ten minute breaks for tea, coffee and light snacks in between. All strips were color-coded by stitching colored cotton ribbons along the hem in order to blind mosquito collectors and field supervisors to their treatment status.

In order to measure the effect of user-occupied treated strips upon nearby non-users, an M-trap was placed at different distances away from a HLC mosquito collector (Fig 2B). Potentially hazardous HLC [25] was only considered justified to apply to the users because it is known that alternative trapping methods can misrepresent the protective effects of repellents for human users [25,26]. One of six predefined distances (2m, 5m, 10m, 20m, 30m and 40m) away from the station where HLC was conducted by the strip user were randomly allocated, without replacement within each one-night randomization cycle, to each of the six two-hour intervals between 18:30 hours and 06:30 hours each night (18:30 to 20:30, 20:30 to 22:30, 22:30 to 00:30, 00:30 to 02:30, 02:30 to 04:30 and 04:30 to 06:30). At the start of each new two-hour period, the M-trap was moved to that allocated distance, along one of 12 pre-defined directions, which was fixed for each night of experimentation. In order to account for balance out, and account for, the highly variable effects of wind speed and direction, these 12 pre-defined directions were evenly distributed 30° apart around the full compass (0°, 30°, 60°, 90°, 120°. 150°, 180°. 210°, 240°. 270°, 300° and 330°, relative to north) and were allocated randomly, without replacement, to each of 12 sequential nights within a 12-night randomization cycle. Pairs of volunteers at each station crossed over between conducting HLC and acting as mosquito baits in the M-traps after every day. However, the allocation of pairs of volunteers to stations within a site remained fixed throughout the study, so that these two causes of variation could be combined into a single-variable source of variance in the statistical analysis.

### Experiment 2: Efficacy of different doses of transfluthrin treated strips

The protective efficacy of different doses of technical grade transfluthrin (all first emulsified by mixing with 90 ml of water and 10ml of liquid detergent as described above) used to treat Hessian strips was determined following a very similar procedure to the one described above for the assessment of long-term efficacy (Fig 1).

The first substantive modification was that these studies were limited to the first half of the night, from 19:00 to 01:00, when *An*. *arabiensis* densities were noticed to be highest. This six-hour period was divided into six one-hour periods (19:00 to 20:00, 20:00 to 21:00, 21:00 to 22:00, 22:00 to 23:00, 23:00 to 00:00 and 00:00 to 01:00), to which was the various distances from the strip and user allocated.

Also the treatment randomization scheme was modified as follows. Two replicate treated strips at each of 8 different doses of transfluthrin, including zero (0, 0.1, 0.2, 0.5 1.0, 2.0, 5.0 and 10ml, with even the zero dosage treatment designated as T for the purposes of relating it to the experimental design description above and the statistical analysis described below), constituting a total of 16 distinct strips. An additional eight replicate control strips, treated with an equivalent mixture of water and detergent only, were selected at random to act as untreated controls (designated as C in the experimental design description above and in the statistical analysis described below) for each site and station on each night. These 16 treated (T) strips and matching untreated control (C) strips were randomly allocated to one of the four sites for a single randomized treatment rotation of three experimental nights, exactly as described above for the efficacy durability assessment. These 48 site-nights of experimentation distributed across 4 sites therefore took 12 nights of experimentation to complete per round of experimental replication. Four rounds of replication were conducted during gaps between the experimental rounds described for the long term evaluation above: The first round was split into two periods because of the extended cool season as described above between the 16^th^ and 26^th^ of July and then the 29^th^ of September until the 11^th^ of October 2014. The second, third and fourth rounds were conducted from the 4^th^ to the 24^th^ of October 2014, from the 17^th^ of November 2014 to the 13^th^ of December 2014, and the 5^th^ to the 24^th^ of January 2015, respectively.

#### Ethics statement

Owners of land on which human landing catches were conducted, provided written consent. All participants provided written informed consent. The participants were provided with drug prophylaxis (Malarone) against malaria [52] and screened weekly for malaria parasites by rapid diagnostic test (mRDT (MAL-Pf, ICT Diagnostics, Cape Town, South Africa, which detects histidine-rich protein II). Fortunately, no participant was found to be infected with malaria during the study but, according to the protocol, any that were would have been offered free-of-charge treatment with artemisinin-lumefantrine, (Co-Artem, Novartis, Basel, Switzerland), the recommended first-line treatment for malaria in Tanzania.

Ethical approvals for all research procedures were obtained from the Institutional Review Board of the Ifakara Health Institute (IHI/IRB/No. 35) and the Medical Research Coordination Committee of the National Institute of Medical Research (NIMR/HQ/R.8c/Vol.ii/125) in Tanzania.

### Storage and use of treated and untreated strips

During experiments, both HLC and M-trap volunteers sat under a shelter made of plastic supported by metal or wooden poles (Fig 2), so that both volunteers and strips were protected from rainfall. During the day, all strips were left hanging on the four poles, in the shade of these shelters where they were protected from rainfall and direct sunlight. During extended breaks in experimental use, strips were left hanging outdoors on wooden poles under a tree at the Ifakara Health Institute (IHI) facilities, under conditions expected to be representative of normal use and storage.

### Mosquito collection and genus identification

Volunteers conducting HLC caught mosquitoes that landed on their legs using a mouth aspirator and a head lamp while those assigned to M-Traps collected mosquitoes from traps, also with a mouth aspirator and head lamp. All mosquito capture by either HLC or M-trap was conducted for either 45 minutes (Dose-response experiments) or 1 hour and 45 minutes (Long term efficacy experiments) and volunteers took 15 minutes break and helped themselves to coffee or tea. All mosquitoes collected in each 1 hour (Dose-response experiments) or 2 hour (Long term efficacy experiments) collection period, at each HLC station or nearby M-trap, were placed in separate labelled paper cups. On each morning after the field experiments, the content of each cup was sorted separately into distinct morphologically-identified mosquito taxa, at genus (*Culex* spp. and *Mansonia* spp.), species group (*An*. *funestus sensu lato*) or species complex (*An*. *gambiae sensu lato*) level. A sub-sample of specimens identified as members of the *An*. *gambiae s*.*l*. complex was taken to the laboratory where identification to sibling species level was carried out by polymerase chain reaction [27]. All data describing the details of the field experimental design, numbers of mosquitoes caught from each sorted taxon, and identified as members of various *An*. *gambiae s*.*l*. sibling species in the laboratory were recorded using the ED1, SS1 and SO1 paper forms recently described for informatically-robust, standardized collection of mosquito data in entomological experiments or surveys [28].

### Experiment 3: Determination of indoor air concentration of transfluthrin released by treated hessian strip

Experiments to measure concentrations of transfluthrin in air were conducted by BioGenius and Dräger Safety AG & Co. KGaA, two independent analytical services companies in Germany (See S1 Supporting Information for the detailed report). A hessian strip exactly like those described above was treated with 1ml of transfluthrin (98.9%, Bayer CropScience AG, Germany), by first suspending in water and detergent in exactly the same way as above, and then cut into four equal pieces. Only one of these pieces was placed in an enclosed room, to avoid overloading the sorbent capacity of the collection tubes used. The rooms each had an internal volume of 30m^3^ (length = 4.69m, width = 2.56m and height = 2.50m) with internal air temperature varying between 21.9 and 24.0°C. These rooms were completely sealed, leaving the keyhole as the only point of air flow, in which two tubes containing 13.6g of Tenax TA adsorbent (Dräger Safety AG & Co. KGaA, Germany) were placed. Air from inside the test-rooms were sucked through the tubes using suction pumps set at an airflow rate of 1.5 l·min^-1^, so that hydrophobic molecules like transfluthrin were adsorbed onto the Tenax TA matrix. The key hole was 96cm above the floor and the strip was placed 1.9m from the floor. The rooms were fitted with an electric fan to improve air circulation, but the fan was faced away from the treated strip. The first set of tubes in the keyhole of each room was collected after 1 hour and the second after 24 hours. The quantity of transfluthrin retained within the tubes was determined by thermal desorption followed by gas chromatography using mass spectrometry detection, in accordance with guideline DIN/ISO 16000–6.

### Data analysis

Data was entered in Microsoft Excel and analysed using R statistical software version 3.1.3, augmented with the *lme4* package for fitting generalized linear mixed models (GLMMs). Separate statistical analyses were conducted for each mosquito taxon that was common in the study area, specifically *An*. *arabiensis*, *Culex* spp., and *Mansonia* spp.

As ≥75% protection against mosquito bites was only observed in the first phase of long-term efficacy studies, lasting 48 weeks since the strips were treated, analysis was restricted to this subset of data, over which period the properties of the strips at least approached the target product profile requirements of outlined for spatial repellents [29]. In order to determine the influence of treated strips upon mosquito biting rates experienced by the user and the non-user at a range of distances from the user, GLMMs of mosquito catch count data for each one or two hour period of the night were fitted for each distinct distance, with the users of treated strips conducting HLC in T_C_-C_T_ or C_T_-T_C_ arrangements considered to be at 0m distance, while users of untreated controlled strips conducting HLC at the opposite station in these same arrangement representing non-users at 80m distance. To estimate the effect of treatment at each distance, the treatment status of the strip in which the user sat in that particular station on that particular night (T_C_ in any T_C_-C_T_ or C_T_-T_C_ arrangement) was treated as a categorical factor in the model, with the catches on users of untreated strips in arrangements with no treated strips (both C_C_ observations in C_C_-C_C_ arrangements) as the reference group and catches on users of untreated strips opposite users of treated strips in the same station on the same night (C_T_ in any T_C_-C_T_ or C_T_-T_C_ arrangement) excluded from the analysis in case any diversion of mosquitoes might increase their exposure so that they do not represent true normal individuals in the absence of any intervention. This possibility of mosquito diversion from the users of treated strips to the users of untreated strips, considered as non-users at 80m distance, was similarly accomplished by changing the contrast and reference groups, treating catches on users of untreated strips opposite users of treated strips in the same station on the same night (C_T_ in any T_C_-C_T_ or C_T_-T_C_ arrangement) as the test group, while the catches on users of untreated strips in arrangements with no treated strips (both C_C_ observations in C_C_-C_C_ arrangements) as the reference group were treated as the reference group. The GLMMs fitted to these mosquito catch data applied a Poisson distribution to these count observations, with observation treated as random effect so that the logarithmic transformation of the Poisson function could normalize these otherwise over-dispersed data. Other random effects included in the models were site, date, and period of the night. Note that the collection method was not included as a variable in any of these models because it was consistently either one or the other for all the data subsets each different model for each different distance (HLC for 0m and 80m or M-trap for 2, 5, 10, 20, 30 and 40m) was fitted to.

No prospective measurements of temperature were recorded at the field site so, after it was noticed in the field that transfluthrin-treated strips were less efficacious at lower temperatures, satellite-derived mean daily air temperature estimates for the study area (longitude 34.688° to 36.563° by latitude -9.524° to -7.619°) were obtained from National Center for Environmental Prediction/National Center for Atmospheric Research with halfway grid points included from mean daily reanalysis since 1948 (http://www.esrl.noaa.gov/psd/data/gridded/data.ncep.reanalysis.html). In order to test for the effect of temperature upon protective efficacy, this variable (Expressed in units as degrees Centigrade) was added as an independent variable to the model described for strip users (considered to be at 0m distance). Similarly testing for the effect of time since treatment upon protective efficacy, was achieved by adding the number of weeks since the strip had been treated to the same model.

The effect of different doses upon the protective efficacy of transfluthrin-treated strips was tested exactly as described above for the primary evaluation of their impact for users and for non-users at various distances, except that non-user data collected with M-traps was excluded from the analysis and different models were fitted for each different dose evaluated.

## Results

The total number of mosquitoes collected was 317,354. They included 51,453 *An*. *arabiensis*, 2,530 *An*. *funestus s*.*l*., 185,822 *Mansonia* spp., 50,233 *Culex* spp., 20,953 *An*. *coustani*, 1,116 *An*. *squamosus*, 1,451 *An*. *wellcomi*, 144 *An*. *pharoensis* spp. and 3,652 *Coquillettidia* spp. mosquitoes.

### Experiment 1: Long-term protection of users and non-users for strips treated with the maximum dose

**Fig 3:**
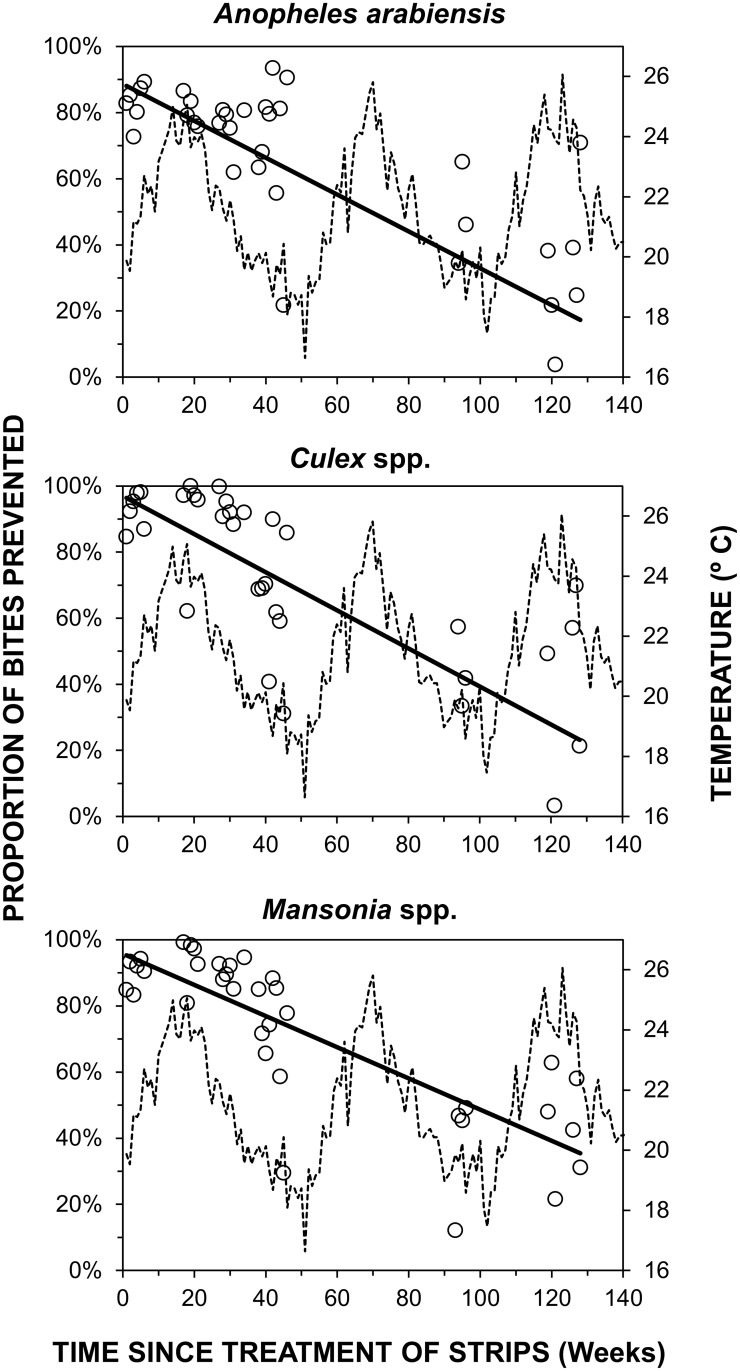
Duration of relative protection conferred by treated hessian strips against biting mosquitoes over time. Weekly aggregate crude estimates of protection against all three of the most common mosquito taxa present over the course of a 2.5 year period following initial transfluthrin treatment of the sacking strips (Fig 1, Experiment 1, Objective 1.1). Estimated mean weekly daily mean temperature is also plotted to illustrate how cold spells were initially observed to be associated with reduced protective efficacy.

**Fig 4:**
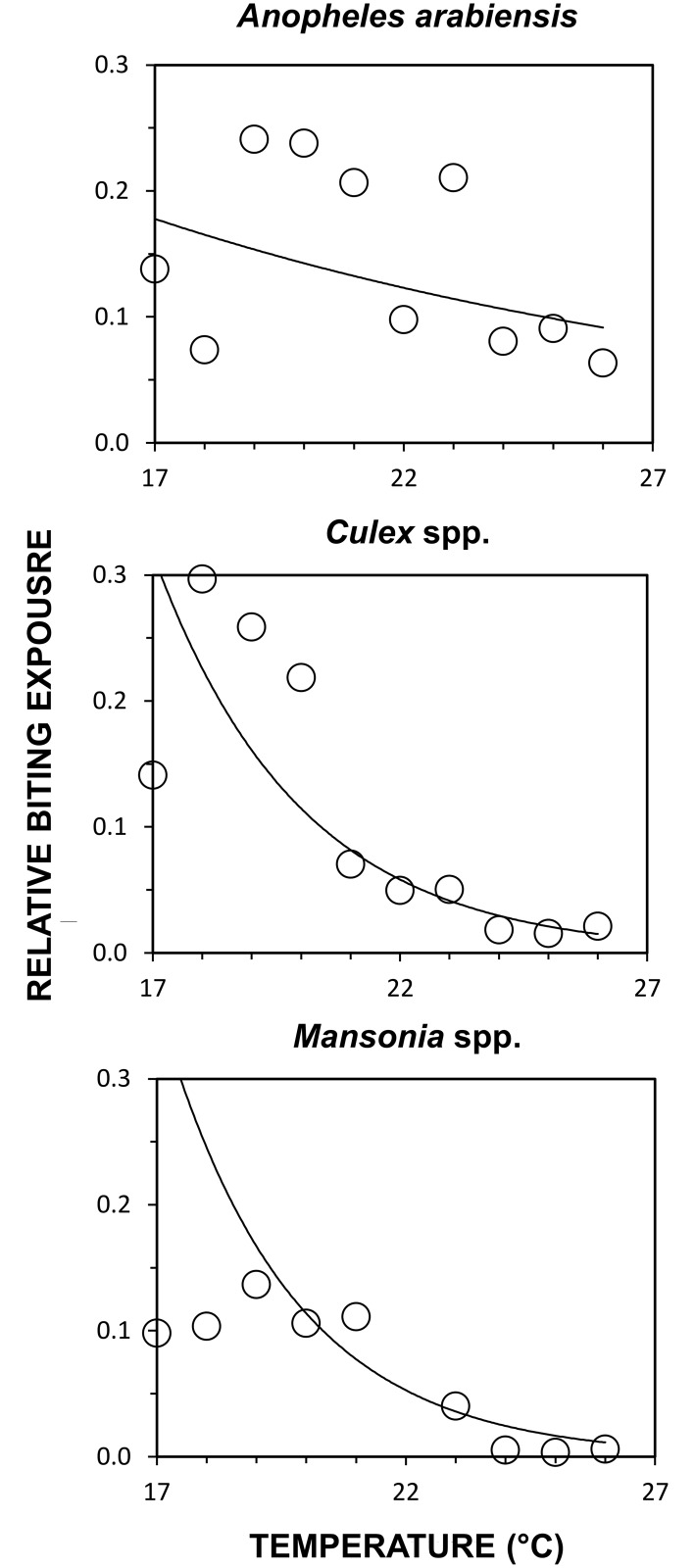
Differential effect of temperature on efficacy of hessian strips against biting mosquitoes. Effect of daily mean temperature for the day preceding each night of experimentation upon efficacy of hessian strips treated with 10ml of transfluthrin, over the first year post-treatment (Fig 1, Experiment 1, Objective 1.1). Each data point represents the mean relative rate of mosquito capture through human landing catches conducted by users of treated strips, averaged within each one degree temperature range category.

Strips treated with 10ml of transfluthrin prevented at least three quarters of outdoor bites by *An*. *arabiensis*, *Culex* spp. and *Mansonia* spp. mosquitoes for at least one year (Relative protection (RP) [95% confidence interval (CI)] = 0.85 [0.71, 0.91], 0.92 [0.78, 0.97] and 0.94 [0.80, 0.98], respectively, P<0.001 in all cases), and conferred significant protection for the entire 2.5 year study duration (Fig 3). Treated strips significantly reduced relative biting exposure to mosquitoes (*An*. *arabiensis*: p < 0.004; *Culex* spp.: p < 0.001 and *Mansonia* spp.: p < 0.001), particularly when preceding mean daily temperatures were greater than 23°C (Fig 4). During the first year, strips prevented more than 90% of bites by all three major mosquito taxa whenever preceding mean daily temperature reached 23°C or more: (RP [95% CI] = 0.91 [0.80; 0.96], p < 0.001) for *An*. *arabiensis*, 0.98 [0.93; 0.99], p < 0.001 for *Culex* spp., and 0.99, [0.99; 0.99], p < 0.001 for *Mansonia* spp.) When data was pooled for all nights over the first year with preceding daily mean temperatures below 21°C, mean protection was nevertheless reasonably satisfactory, at approximately 80% for *An*. *arabiensis* (RP [95% CI] = 0.80 [0.63; 0.90], p < 0.001) and *Culex* spp. (RP = 0.80 [0.48; 0.93], p < 0.0001), and closer to 90% for *Mansonia* spp. (RP [95% CI] = 0.89 [0.89; 0.89], p < 0.001).

**Fig 5:**
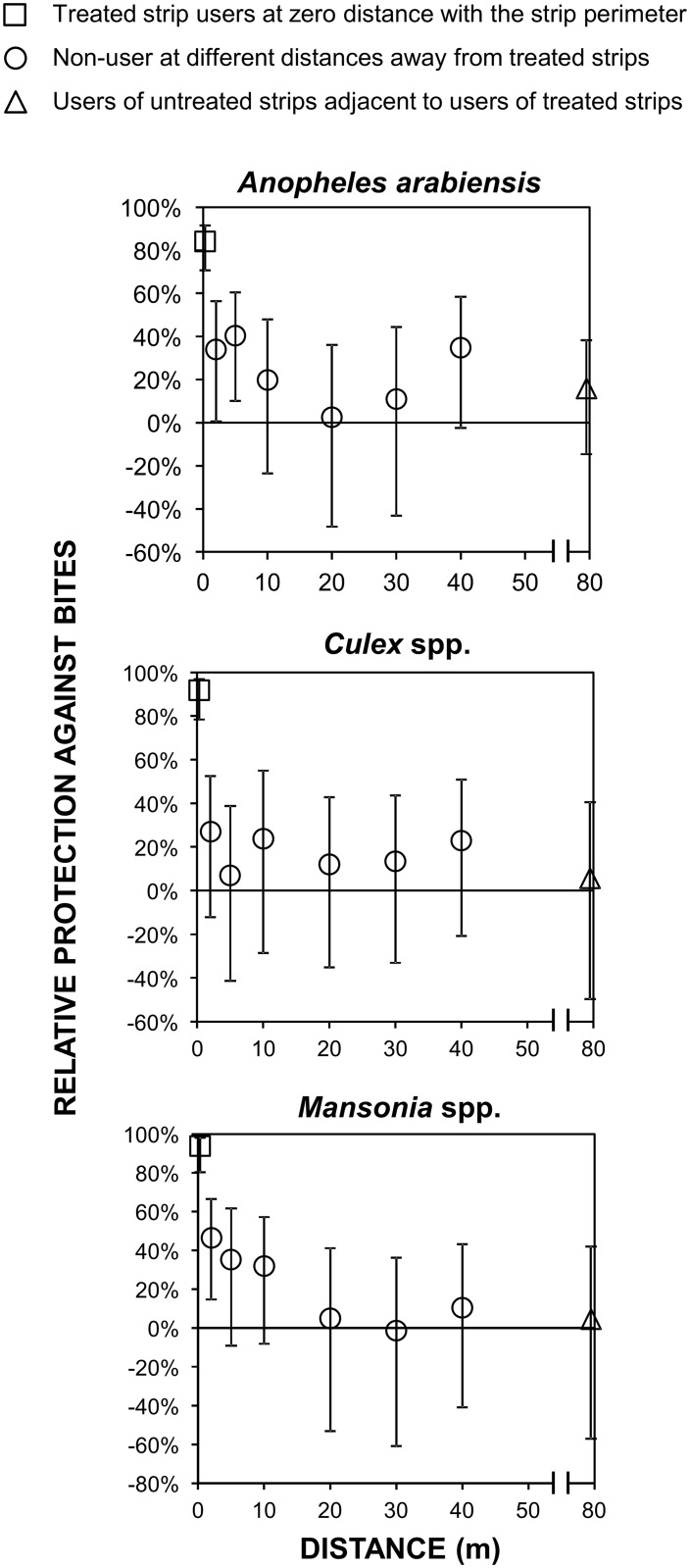
Effect of treated hessian strips upon human exposure to mosquitoes as a function of distance. Estimated proportional protection of users and nearby non-users of treated strips (Fig 1, Experiment 1, Objectives 1.2 and 1.3). This analysis was restricted to data from the first year of experiment 1 (Fig 3), for which strips were treated with the maximum 10ml dose.

In addition to protecting the actual users sitting within their perimeter (Fig 2A), strips treated with the same 10ml dose evaluated in previous studies [17,18] even provided almost 50% protection against *An*. *arabiensis* (RP [95%CI] = 0.42, [0.34, 0.49], p < 0.001) to non-users (humans acting as baits in M-traps) located within 5m of them (Fig 5). However, no significant protection of non-users, even at only 2m distance, was observed for *Culex* spp. or *Mansonia* spp. (Fig 5). Reassuringly, no evidence of diversion of mosquitoes from any major taxa from users to non-users was observed at any distance evaluated up to 80m radii (Fig 5).

### Experiment 2: Dependence of protection upon transfluthrin dose

**Fig 6:**
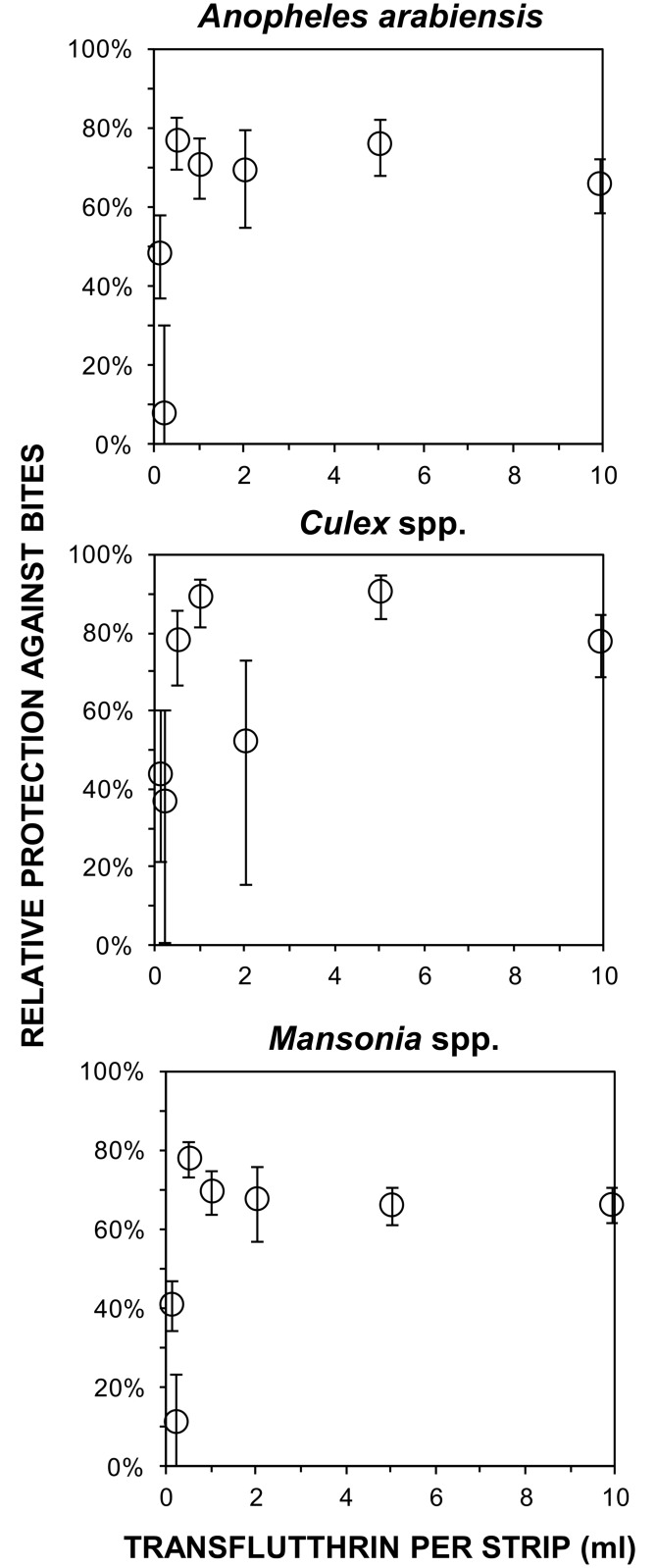
Protective efficacy of different doses of transfluthrin treated hessian strips. Relative proportional protection of users of strips treated with different doses of transfluthrin (Fig 1, Experiment 2, Objective 2).

As little as 1ml of transfluthrin provided equivalent protective efficacy to strips treated with the 10-fold higher dose previously evaluated, and no increase in protective efficacy was observed as dosage was increased beyond that minimum required level (Fig 6). For instance 1ml transfluthrin treated strips reduced biting exposure to *An*. *arabiensis* (RP [95%CI] = 0.71 [0.62, 0.78], p < 0.001) to the same degree as the 10ml dose (RP [95%CI] = 0.66 [0.58, 0.73], p < 0.001).

### Experiment 3: Indoor air quantity of transfluthrin released by a hessian strip

The quantity of transfluthrin collected within 1 hour from the rooms with treated strips inside was below the detection threshold of 2ng per tube. After 24 hours the quantities of transfluthrin recovered from tubes were 623ng, 1370ng and 863ng from each room, corresponding to a mean (± standard error) concentration in air of only 0.13 ± 0.06 μg·m^-3^, which is >1000 times lower than the maximum acceptable exposure concentration for long-term inhalation exposure of human beings of 500 μg·m^-3^, as defined by the regulatory authorities of the European Union (EU) [30].

## Discussion

**Fig 7:**
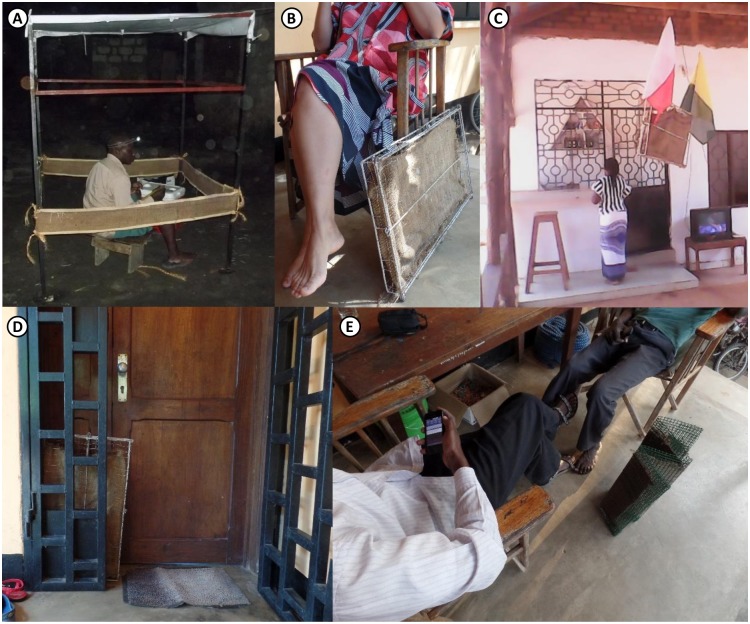
Examples of the rapidly diversifying array of physical formats for hessian strip transfluthrin emanators derived from the prototype evaluated here. **A**: The previously described lead prototype emanator [17,18] which was evaluated in detail in this manuscript. **B**: A second, more convenient “flag” prototype [31], with the fabric strip enclosed in a welded iron frame, leaning up against a user’s chair on her porch. **C**: The same second flag prototype hanging in a public bar [31]. **D**: The same second prototype placed up against an end user’s front door, to prevent house entry by *Culex* spp. mosquitoes which rest upon the door and then enter when it is opened in the morning. **E**: The third and latest, foldable, self-standing prototype, in which the hessian strip is protected from moisture on the ground, and users are protected from physical contact with the active ingredient, by sandwiching it within a wire mesh cover with wide margins.

This study demonstrated that hessian strips treated with 10ml of transfluthrin reduced exposure to vectors of lymphatic filariasis, several arboviruses and malaria by more than three quarters for one year after treatment, and continued to provide some degree of protection for over two years. These results highlight the potential of such low-technology crude fabric emanators for integrated, simultaneous protection against multiple mosquito species that exhibit outdoor biting behavior, overlap geographically, and co-transmit multiple disease pathogens. Control tools that target more than one vector maximize disease-control benefits and are therefore more cost effective than those targeted against single specific species [1]. While more practical formats for routine use of these fabrics in everyday life in a range of settings (Fig 7) will need to be fully developed and evaluated, and existing systems for bed net delivery and retreatment will need to be adapted to the specific requirements of this technology, it is nevertheless encouraging that this prototype has such high efficacy for so long and can be safely formulated anywhere with minimal safety precautions in the same way as an LLIN retreatment kit.

The most obvious limitation of the emanator prototype evaluated here is its somewhat awkward format (Fig 7A) which may well preclude routine use during everyday life [7,18]. However, studies have demonstrated that these fabric strips can be crafted into a range of far more practical prototypes, such as decorative baskets, flags, curtains, wall hangings, some of which are illustrated in Fig 7. Indeed, transfluthrin-treated hessian decorative baskets and flags have already been tested and have proven efficacious against outdoor biting mosquitoes in representative deployment settings, specifically as outdoor bars [31].

Crude natural fiber fabrics are widely available across the tropics and transfluthrin is available in bulk from generic sources, both at affordable prices that are comparable with bed nets and bed net treatments. One of the hessian sacks made of jute that were used here to make each of the strips assessed here cost approximately US$3.30, and other locally-available crude cellulose fabrics, such as sisal or hemp, may well be similarly suitable. While this 1ml dosage of transfluthrin (1.5g) is considerably greater than that used in equivalent proprietary products containing metofluthrin (0.2g), the former generically-manufactured active ingredient is far more readily available and affordably priced: Transfluthrin is available in bulk at market prices ranging from $100 to $200 per kilogram so the 1 ml minimum dose that was fully protective for 6 months under these field conditions (Fig 6) would cost less than $0.20 per treatment.

The exact mechanisms of action of a wide variety of insecticides generically referred to as repellents are diverse and controversial [29,32,33], because of the risk of diversion to unprotected non-users nearby [11,34]. Topical repellents reduce human-vector contact and protect users against bites but unfed mosquitoes are diverted from users to non-users [11,34]. Reassuringly, treated strips not only reduced exposure to mosquito bites up to 5m away from the user, there was also no evidence of diversion to nearby unprotected non-users at any distance within an 80m radius from treated strips.

These observations are consistent with recent studies showing that, transfluthrin incapacitates mosquitoes by inhibiting blood feeding [35], rather than repelling them in the strict sense [33,36]. Furthermore, transfluthrin can also kill mosquitoes that closely approach protected humans [6], which would also preclude diversion to unprotected individuals nearby. Transfluthrin-treated hessian emanators may therefore be far safer for nearby non-users than conventional topical repellents like N,N-Diethyl-meta-toluamide (DEET) [11,34] and might even provide area-wide protection of users and non-users alike, similarly to LLINs and IRS [12,35]. Given the limited number of tools available for protecting against outdoor-biting mosquitoes, and the limited durability of existing repellent products, the observed reduction of biting exposure by more than three quarters over a period of almost a full year, without retreating the strips, is both unprecedented and encouraging. While killing adult mosquitoes is considered to have the greatest impact upon disease transmission, reducing human biting exposure by deterring host-seeking mosquitoes can nevertheless have valuable impacts upon transmission, arising from mass effects upon the size, human-feeding frequency and longevity of entire mosquito populations [37].

Nevertheless, this study only evaluated efficacy at individual level, in terms of reduced mosquito biting pressure, and it is only possible to speculate about which mechanism or mechanisms of action were responsible for the personal protection observed here. It will therefore be important to conduct cluster-randomized studies, with both entomological and epidemiological outcome indicators, to determine whether reduced disease transmission to nearby non-users, and indeed entire communities, can also be achieved [38]. However, before proceeding to full-scale cluster-randomized studies, it may be prudent to directly compare this new technology with the most promising available alternatives, such as proprietary passive metofluthrin emanators [13,14,15,16,39], in terms of simple entomological indicators of biting exposure like those used here [40,41].

The pyrethroids class of insecticides, to which transfluthrin belongs, is currently the most widely-used, safest and cost-effective chemical class of insecticides available for public health applications. Furthermore, it is the only insecticide class considered safe enough for personal protection applications involving direct human contact with bed nets, hammocks or clothing [42]. Unfortunately, the rate at which mosquitoes are becoming resistant to pyrethroids is alarming and represents an immediate, substantive threat to recent public gains made in the control of mosquito-borne diseases, malaria in particular [43,44]. It has been suggested that the tetrafluorobenzyl moiety of transfluthrin may make it more efficacious against some populations of pyrethroid-resistant mosquitoes than the conventional, non-volatile pyrethroids used for LLINs, IRS and impregnated clothes [45]. However, distinct metabolic, knock-down and cuticular forms of resistance against pyrethroids have already been documented and more may emerge, [43,44] so it seems unlikely that the polyflurobenyl-modified pyrethroids alone will provide a panacea to address this “looming public health catastrophe” [43]. Furthermore, recent studies of *Aedes aegypti* indicate that physiological resistance to the lethal effects of transfluthrin may be associated with concomitantly decreased responsiveness to its repellent mode of action at sub-lethal doses [46].

While the susceptibility of *An*. *arabiensis* in this setting to three different conventional, solid-phase pyrethroids was assessed over the period of this study [20], no measurements of susceptibility to transfluthrin itself were conducted for any of the three common mosquito taxa these emanators were evaluated against. Looking forward, it would therefore be important to assess susceptibility of target mosquito populations to the lethal, repellent and incapacitating properties of transfluthrin using standardized, controlled laboratory assays in future studies. Furthermore, experimental hut studies [47,48] would be invaluable to measure the mortality and deterrence rates of wild mosquitoes exposed to transfluthrin vapor delivered through this emanator format under field-relevant conditions, to examine how these distinct modes of action are affected by existing mechanisms of resistance to pyrethroids, and determine whether these mechanisms are co-associated or independent of each other. While the resistance status of the *Culex* spp. and *Mansonia* spp. populations in this setting at the time of the study was not determined, it is encouraging that the *An*. *arabiensis* populations these transfluthrin emanators proved so efficacious against were clearly resistant to all three conventional, solid-phase pyrethroids they were tested against in 2014 [20], when the dose-response studies described in Fig 6 were conducted. Furthermore, the >90% protection obtained against all three mosquito taxa on nights with high air temperatures (Fig 4) are consistent with previous evaluations in the warmer coastal climate of Dar es Salaam, where >90% protection against both *An*. *gambiae* and *Culex* spp. was observed for almost four months [18]. It therefore appears that the slightly lower levels of protection reported here from an inland setting with greater seasonal temperature variation arose from efficacy limitations caused by cooler conditions and lower transfluthrin evaporation rates, rather than any lack of responsiveness of local mosquito populations.

While further studies of potential for human inhalation, dermal and oral exposure to transfluthrin under conditions of routine use would be invaluable, the long-established safety record of transfluthrin, and the low but nevertheless efficacious vapor concentrations reported here, are reassuring. Even under the worst-case scenario conditions of indoor use in an enclosed room with negligible ventilation, the estimated level of human exposure to airborne transfluthrin vapor is more than three orders of magnitude below the maximum acceptable long-term exposure concentration defined by regulatory authorities in the EU [30]. Indeed the EU lists transfluthrin in Annex I of the Biocidal Products Regulation [30], meaning that it is classified along with food and cosmetic additives, pheromones and other substances that are considered to have such low toxicity that they are eligible for simplified authorization procedures [49].

An additional considerable limitation of this study is that equivalent measurements of transfluthrin vapor concentration were not undertaken outdoors during the field-based efficacy assessments. Such studies should be prioritized in the future, so that the way in which this airborne insecticide affects mosquito behavior can be far better understood, by quantifying the loading doses and resulting airborne concentrations required to elicit specific mosquito responses. It will also be essential to quantify the release rate of the active ingredient from a given absorbent substrate, as well as the decay rates of retention within the fabric, to determine half-life and retreatment frequency for the device, under a range of different environmental conditions.

### Conclusions

Despite these study limitations, the experiments reported here clearly demonstrate how transfluthrin-treated hessian emanators can provide safe, affordable, long-term protection against three of the most medically important mosquito taxa in the world, one of which was known to be resistant to conventional, solid-phase pyrethroids at the time [20]. Crucially, all the protective efficacy measurements reported in Figs 3 to 6 were conducted outdoors, where active humans cannot be protected by bed nets or residual sprays with conventional, solid-phase insecticides. In addition to the long-standing problems of outdoor transmission of a range of mosquito-borne pathogens through multiple vectors [1], the recent rapid spread of pandemic urban Zika virus transmission from human to human [50] via several potential vectors that often bite outdoors now merits urgent attention [51,52,53]. Further studies to adapt this prototype to formats suitable for programmatic scale-up, and to assess their impacts and mechanisms of action when applied at high coverage across entire populations, should therefore be immediately prioritized.
